# Discrepancies in Assessing Diastolic Function in Pre-Clinical Heart Failure Using Different Algorithms—A Primary Care Study

**DOI:** 10.3390/diagnostics10100850

**Published:** 2020-10-20

**Authors:** Martina Setti, Giovanni Benfari, Donato Mele, Andrea Rossi, Piercarlo Ballo, Maurizio Galderisi, Michael Henein, Stefano Nistri

**Affiliations:** 1 Division of Cardiology, Department of Medicine, University of Verona, 37126 Verona, Italy; martina.setti@hotmail.com (M.S.); giovanni.benfari@gmail.com (G.B.); andrea9rossi@gmail.com (A.R.); 2 Department of Cardiac, Thoracic, Vascular Sciences and Public Health, University of Padova, 35128 Padova, Italy; donatomele@libero.it; 3 Santa Maria Annunziata Hospital, Cardiology Unit, 50012 Florence, Italy; pcballo@tin.it; 4 Department of Advanced Biomedical Sciences, Federico II University Hospital, 80131 Naples, Italy; mgalderi@unina.it; 5 Institute of Public Health and Clinical Medicine, Umeå University, 90187 Umeå, Sweden; michael.henein@umu.se; 6 CMSR Veneto Medica-Cardiology Service, 36077 Altavilla Vicentina (VI), Italy

**Keywords:** diastole, guidelines, echocardiography, primary care, pre-clinical heart failure

## Abstract

Background: Current guidelines on diastolic function (DF) by the American Society of Echocardiography and the European Association of Cardiovascular Imaging (ASE/EACVI) have been disputed and two alternative algorithms have been proposed by Johansen et al. and Oh et al. We sought (a) to assess the concordance of ASE/EACVI guidelines on DF using these proposed alternative approaches and (b) to evaluate the prevalence of indeterminate diastolic dysfunction (DD) by each method, exploring means for reducing their number. Methods: We retrospectively analyzed the echocardiographic reports of 1158 outpatients including subjects at risk of heart failure without (*n* = 644) or with (*n* = 241) structural heart disease, and 273 healthy individuals. Concordance was calculated using the k coefficient and overall proportion of DD reclassification rate. The effectiveness of pulmonary vein flow (PVF), Valsalva maneuver, and left atrial volume index/late diastolic a’-ratio (LAVi/a’) over indeterminate grading was assessed. Results: The DD reclassification rate was 30.1% (k = 0.35) for ASE/EACVI and OH, 36.5% (k = 0.27) for ASE/EACVI and JOHANSEN and 31.1% (k = 0.37) for OH and JOHANSEN (*p* < 0.0001 for all comparisons). DF could not be graded only by ASE/EACVI and OH in 9% and 11% patients, respectively. The majority of patients could be reclassified using PVF or Valsalva maneuver or LAVi/a’, with the latter being the single most effective parameter. Conclusion: Inconsistencies between updated guidelines and independent approaches to assess and grade DF impede their interchangeable clinical use. The inconclusive diagnoses can be reconciled by conventional echocardiography in most patients, and LAVi/a’ emerges as a simple and effective approach to this aim.

## 1. Introduction

The assessment of left ventricular (LV) diastolic function (DF) is an integral part of a comprehensive echocardiographic examination. Over the years, multiple guidelines and independent algorithms have been developed and validated. However, the GRAding Diastolic dysfunction in Outpatients (GRADO) Study [1] demonstrated substantial differences when concordance was assessed between three documented algorithms, despite the high feasibility of DF measurements [2,3,4]. Moreover, grading diastolic dysfunction (DD) was not achievable in 6 to19% of individuals using those methods. Furthermore, using a large number of variables, according to the American Society of Echocardiography (ASE)/European Association of Cardiovascular Imaging (EACVI) 2009 algorithms [4], did not prove practical for real-world clinical arena.

The 2016 joint ASE/EACVI guidelines attempted to simplify the assessment of LVDF in clinical practice [5]. Despite hemodynamic and prognostic validations of the 2016 ASE/EACVI guidelines [6], some potential limitations were noticed in their clinical application. The resulting debate generated the proposal of two other approaches. Johansen et al. [7] suggested a 2-step approach in which the normality of e’ precluded the presence of DD while low e’ values were consistent with DD, which grading was obtained by additional evaluations summing 3 further parameters. Oh et al. [8] proposed a unifying approach starting from the same 4 parameters employed by 2016 ASE/EACVI guidelines (see methods).

Due to the significant burden imposed by heart failure (HF) on the health system, patients and care-providers, the American College of Cardiology (ACC) and American Heart Association (AHA) released an HF staging system emphasizing identification of asymptomatic patients with clinical risk factors for HF without (Stage A—SAHF) or with (Stage B—SBHF) cardiac structural and functional abnormalities [9]. Since asymptomatic LVDD is associated with incident HF and reduced survival and quality of life, it has been advocated to be included in SBHF [10,11]. However, in a busy primary-care facility, where these patients are usually evaluated, the application of complex algorithms and newer, advanced techniques (i.e., left atrial (LA) strain/speckle tracking) may be cumbersome and largely unpractical.

Thus, we planned the present study to assess the discrepancies among the 2016 ASE/EACVI guidelines and the two previously cited alternative algorithms [7,8] in two cohorts of healthy, adult individuals (stage 0 HF: S0HF) [12], and SAHF and SBHF patients [1]. In fact, interchangeability and agreement of different classifications are clinically relevant in daily practice, both in decision making and for serial examinations of individual patients. We also sought to assess the effectiveness of standard Doppler and tissue Doppler imaging (TDI) methods in classifying patients with undetermined patterns. Moreover, we explored the potential role of combining LA volume index (LAVi) measurement and cavity function assessed by late diastolic TDI a’ wave (LAVi/a’ ratio) since this approach was previously used in identifying advanced DD [13,14,15].

## 2. Materials and Methods

### 2.1. Study Population

We retrospectively analyzed the echocardiographic data of 2 different study groups. The first one was composed by healthy individuals aged > 35 years [12], including athletes and non-athletes (S0HF). The second cohort was formed by outpatients, free from prevalent or incident HF, in sinus rhythm, without electric, myocardial, or rhythm disturbances that could influence the natural pattern of diastolic measurements [1]. Based on clinical records, these patients were subsequently classified as SAHF if they were at increased risk of HF for the presence of predefined clinical risk factors, in the absence of structural heart disease (reduced LV ejection fraction (LVEF), regional wall motion abnormality, LV enlargement based on LV end-diastolic volume indexed to body surface area (BSA), LV hypertrophy (LVH) based on gender-adjusted criteria of LV mass indexed to BSA) or as SBHF if those structural heart abnormalities were present [9]. Based on the original study designs [1,12], patients with any degree of mitral stenosis, more than mild aortic or other mitral valve disease, and severe mitral calcifications were excluded.

### 2.2. Echocardiography

LV size and function were assessed according to current recommendations [1,4,12]. LAVi was obtained from dedicated views and indexed to BSA using bi-plane area length method [16]. All indexes for assessing DF were measured from the apical views. Mitral inflow peak early (E) and late (A) velocities, E/A ratio, E wave deceleration time (DT), and A wave duration (A_dur_) were measured at baseline and during the strain phase of the Valsalva maneuver, in all SAHF and SBHF patients. In the same patients’ groups, pulmonary vein peak systolic (S) and diastolic (D) flow velocities, S/D ratio, atrial reversal flow (AR) velocity and the duration of flow during atrial contraction (AR_dur_) were recorded. Using pulsed-wave TDI, mitral annular velocities (peak systolic (s’), early diastolic (e’), and late diastolic (a’)) were measured at the two annular sites (septal and lateral) and averaged. The E/e’ ratio was also calculated [1,12]. Tricuspid regurgitation (TR) jet peak velocity (TRV) was determined from multiple views and dichotomized based on the threshold value of 2.8 m/s. When the quality of TR envelope did not allow optimum measurement, additional echocardiographic criteria were used to assign the probability of pulmonary hypertension [17]. These patients were considered equivalent to TR ≤ 2.8 m/s if they were diagnosed at low probability of pulmonary hypertension, whilst the remaining (intermediate or high probability of pulmonary hypertension) were considered as having TR > 2.8 m/s.

### 2.3. Diastolic Function Algorithms

ASE/EACVI 2016 recommendations [5] were used paying particular attention to the absence of myocardial abnormalities (pathological LVH, wall motion abnormalities) to allocate patients with normal LVEF to the first algorithm (A-algorithm), which includes four variables (septal and lateral e’, the average E/e’ ratio, LAVi, and TRV) with appropriate thresholds. If more than 50% of criteria existed (3 out of 4 or 2 out of 3 available variables), the patient was considered to haveDD, and the B-algorithm was then used to grade it. In the A-algorithm, patients in whom half of the available criteria did not meet the cut-off values were classified as indeterminate. Patients with reduced LVEF and/or any of the previous myocardial abnormalities were directly analyzed by the B-algorithm using the mitral inflow E/A ratio as well as E-wave velocity. Grade I DD with normal LV filling pressure (LVFP) was determined if patients had E/A ratio < 0.8 and E-wave peak velocity < 50 cm/s. Patients with E/A > 2 (restrictive pattern) were labelled as grade III DD, with raised LVFP. Patients with intermediate filling patterns (0.8 < E/A < 2) or with E/A < 0.8 but E-wave peak velocity > 50 cm/s, underwent a further analysis based on 3 criteria (average E/e’ > 14, TRV > 2.8 m/s and LAVi > 34 mL/m^2^). Patients with at least 2 criteria were classified as grade II DD with increased LVFP, and grade I with normal LVFP was attributed to those with at least 2 negative criteria. Patients in whom there was discrepancy between 2 available parameters and those in whom only one parameter was available were labelled “cannot determine LVFP and DD grade”.

The algorithm proposed by Johansen et al. (referred to as JOHANSEN) [7] pivots on the average e’ velocity, using a cut-off value of 9 cm/s, above which patients are considered to have no DD. Patients with low values of e’ (<9 cm/s) were otherwise considered to have DD, which grading was obtained by summing 3 further parameters: (a) E/A ratio > 2; (b) E/e’ ratio > 13, and (c) LAVi > 34 mL/m^2^. The presence of 1, 2 or all 3 of these criteria resulted into a 3-tier grading.

The method proposed as a revised, unified algorithm by Oh et al. (referred to as OH) [8] used as initial step the same 4 variables of the ASE/EACVI, with the noticeable difference of employing only septal TDI measures and, consistently, appropriate threshold values, i.e., septal e’ < 7 cm/s and septal E/e’ > 15. This method provided strict criteria (presence of at least 3 out of 4 criteria) as further gate-keeper, patients were subsequently classified based on E/A ratio as normal DF or grade I DD if ≥3 first-step variables were absent and E/A was >0.8 or ≤0.8, respectively. If ≥3 first-step variables were present, Grade II or III DD was assigned if E/A <2 or ≥2, respectively. If patients had only 2 first-step variables, they described as ‘undetermined DF’.

### 2.4. Assessment of Undetermined DF

Multiple variables have been suggested to clarify the hemodynamic condition of the patients described as having undetermined DF [5,8]. Some of these parameters are cumbersome and need specific advanced technological tools which make them unfeasible in primary care setting [18].

Other parameters are more feasible such as the quantitative analysis of pulmonary vein flow (PVF) and of mitral inflow during Valsalva maneuver. Indeed, the difference between AR_dur_ and A_dur_ was calculated, with an AR_dur_–A_dur_ > 30 ms as a reliable marker of increased LV end-diastolic pressure, independent of LVEF [19]. A relative decrease of 50% or an absolute decrease of 0.5 in the E/A ratio, based on each classification, were used as a highly specific feature for raised LV filling pressures. In addition, we decided to explore the potential role TDI a’, alone [20,21,22] or in combination with LAVi (LAVi/a’) [13,14,15] in re-classifying patients in the undetermined group.

### 2.5. Statistics

Continuous variables were expressed as mean ± standard deviation. Categorical variables were presented as absolute numbers and percentage. Differences between groups were analyzed using the Student *t*-test for independent groups or the Chi-square test, as appropriate. Correlations between variables were evaluated using the Pearson or Spearman’s coefficients. Linear regression analysis was used to test the association between LAVi/a’ or a’ and DD grades. The variable with the best model fitting was selected to further stratify patients with undetermined DF. In these patients, we subsequently identified two groups: those reclassified using traditional parameters (PVF and Valsalva maneuver) and those who were not reclassified. Receiver operating curves were then built to select an appropriate threshold for the newly proposed parameters (a’ or LAVi/a’), using reclassified vs. not reclassified as target.

The three different approaches for the determination of DD were compared. The concordance between different approaches was tested by analyzing the kappa coefficient and was defined as slight (k 0–0.20), fair (k 0.21–0.40), moderate (k 0.41–0.60), good (k 0.61–0.80), and optimal (k 0.81–1). The overall agreement was measured as the proportion of patients identically classified between two algorithms. The reclassification rate was calculated as the difference between 100% and the proportion of agreement. All analyses were performed using JMP version 14.1.0 statistical software (SAS Institute Inc., Cary, NC, USA). *p* < 0.05 was taken as statistical significance.

## 3. Results

The study population consisted of 1158 individuals, with significant differences in demographic and echocardiographic characteristics across HF stages (Table 1). Seventy-six S0HF subjects (28%) were competitive athletes. Specific clinical characteristics of SAHF and SBHF, including risk factors, renal function, and therapy are in Table A1.

**Figure 1:**
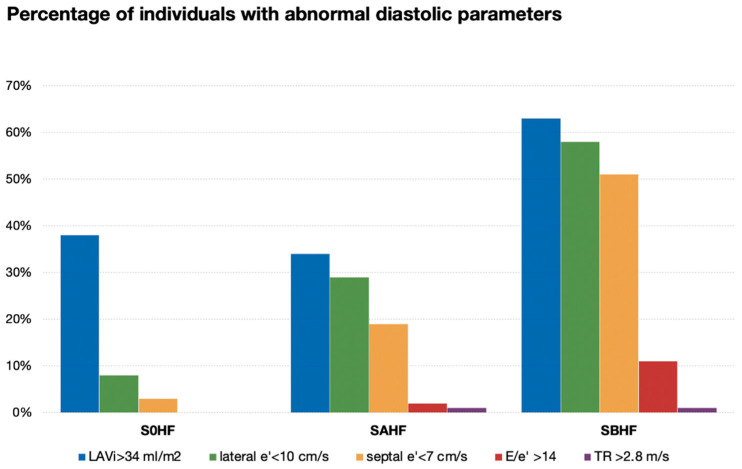
Percentage of patients with each marker of diastolic dysfunction in healthy individuals (S0HF) and patients at risk of heart failure (SAHF, SBHF). Abbreviations: LAVi, Left Atrial Volume index; e’, peak early annular diastolic velocity; E/e’, ratio between E wave velocity and early diastolic velocity of the mitral annulus; TR, tricuspid regurgitation.

Among the basic markers of DD, dilated LAVi (>34 mL/m^2^) was the most frequently present in all stages, followed by reduced e’, high E/e’, and raised TR velocity (Table 1, Figure 1). High E/e’ and raised TR velocity were absent in S0HF. LVH was very prevalent in SBHF (*n* = 211, 88%), but rare in S0HF (*n* = 14). The latter subjects were senior (54 ± 10.2 years), mostly male (57%), competitive athletes, with mild LVH (118.0 ± 18.7 g/m^2^), mildly dilated LV (75.9 ± 22 mL/m^2^), and dilated LA (43.7 ± 13.1 mL/m^2^). TDI indices of DF were normal (average e’12.0 ± 2.8 cm/s and E/e’ 6.8 ± 1.2); per-study protocol [12], they all had normal 12-lead electrocardiogram and were all eligible for competitive sports.

**Figure 2:**
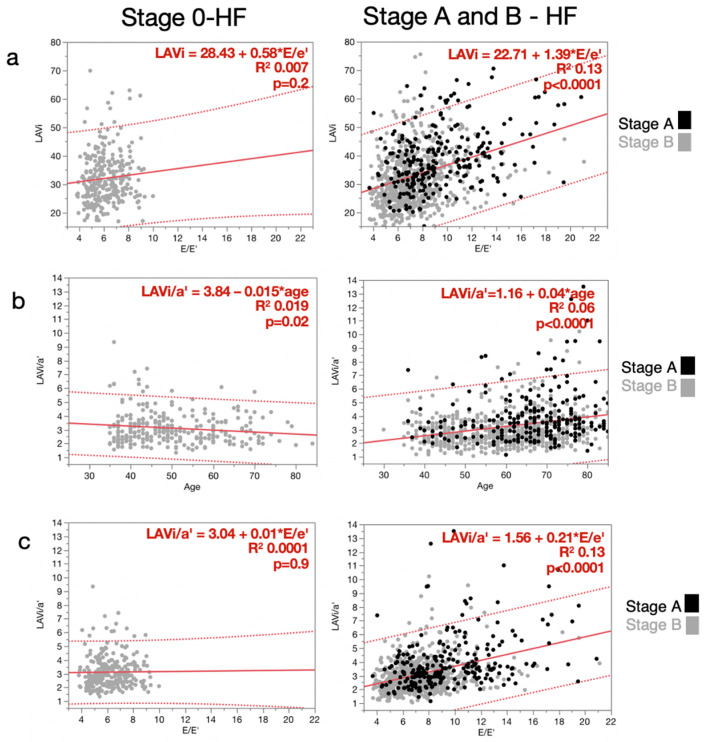
Relationships between LAVi and E/e’ (**a**) LAVi/a’ and age (**b**) and LAVi/a’ and E/e’ (**c**) for healthy individuals (Stage 0 Heart Failure, left), and patients at risk of heart failure (Stage A and B Heart Failure, right). Abbreviations: LAVi, Left Atrial Volume index; E/e’, ratio between E wave velocity and early diastolic velocity of the mitral annulus; LAVi/a’, ratio between Left Atrial Volume index and peak late annular diastolic velocity.

LAVi was marginally related to age in S0HF, particularly in SAHF and SBHF. LAVi was not related to E/e’ in S0HF but related in SAHF/SBHF (Figure 2a). E/e’ and a’ were directly related to age in healthy individuals and in those at risk of HF, while e’ was inversely related to age in both. LAVi/a’ was related to age in S0HF and in SAHF/SBHF, though the relationship was weak. The slope of this relationship was, however, opposite for healthy subjects and SAHF/SBHF patients (Figure 2b). Finally, LAVi/a’ was not related to E/e’ in S0HF, but it was directly related to E/e’ in SAHF and SBHF (Figure 2c).

### 3.1. ASE/EACVI

Applying this method in the 273 S0HF individuals we used the A-algorithm also for the 14 athletes with LVH recognizing the adaptive and physiologic nature of the increased LV mass in these individuals. Normal DF was detected in 263 (96%), while DD was undetermined in 10 (4%). Six hundred forty-four SAHF patients were analyzed by the A-algorithm, 10 of whom with DD, were subsequently evaluated also by B-algorithm. This resulted in 546 (85%) individuals with normal DF, 6 (1%) with grade II DD, and 92 with undetermined DD. Since the 241 SBHF patients were all classified as having “myocardial disease”, they were assessed by the B-algorithm. Two hundred and nine patients (87%) were classified as grade I, 21 (9%) as grade II, and 11 (4%) as grade III DD.

### 3.2. JOHANSEN

Among the S0HF subjects, 254 (93%) were considered to have normal DF, 10 (4%) with mild DD and 9 (3%) with moderate DD. Four-hundred forty-eight SAHF individuals (70%) were diagnosed with normal DF, while the remaining with DD; mild in 98 (15%), moderate in 86 (13%), and severe in 12 (2%). Eighty-five (35%) SBHF patients had normal DF, mild DD in 35 (14%), moderate in 86 (36%), and severe in 35 (15%) SBHF subjects.

### 3.3. OH

Two-hundred fifty-nine S0HF individuals (95%) were recognized as having normal DF, while Grade I DD was present in 8 (3%). Increased filling pressure was not detected in any individual, and DD could not be graded in 6 (2%). Among 644 SAHF patients, DF was normal in 463 (72%) and mildly abnormal in 111 (17%), Grade II and III were present in 12 (2%) and 1 (0.1%), respectively. Undetermined DF was attributed to 57 (9%) patients. In SBHF, DF was normal in 107 (44%) patients and mildly abnormal in 38 (16%). Grade II DD was present in 32 subjects (13%), while grade III DD was detected in 2 (1%). DD could not be graded in 62 (26%).

### 3.4. Concordance Among Methods

**Figure 3:**
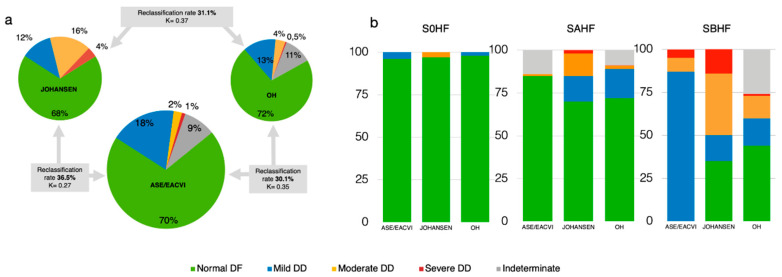
Frequency of different grades of diastolic dysfunction per each algorithm. (**a**) Central pie describes prevalence of different grade of diastolic function based on ASE/EACVI, while, on upper left and right, those for JOAHNSEN and OH are reported, respectively. Reclassification rate and Kappa coefficients are depicted for each comparison. (**b**) Prevalence of grades of diastolic dysfunction per clinical stages, for each method, are represented as pile histograms. Abbreviations: S0HF, Stage 0 Heart Failure; SAHF, Stage A Heart Failure; SBHF, Stage B Heart Failure; DF, Diastolic Function, DD, Diastolic Dysfunction; NL, Normal; DD1, Diastolic Dysfunction Grade I; DD2, Diastolic Dysfunction Grade II; DD3, Diastolic Dysfunction Grade III; IND, Indeterminate.

Among the overall 1158 individuals, comparing ASE/EACVI and OH, the reclassification rate for DD diagnosis was 30.1% (349 patients, k = 0.35 [CI 0.30–0.40], *p* < 0.0001), which rose to 36.5% (423 patients, k 0.27 [CI 0.23–0.30], *p* < 0.0001) when the concordance between ASE/EACVI and JOHANSEN was analyzed. When OH and JOHANSEN algorithms were compared, the reclassification rate was 31.1% (360 patients, k 0.37 [CI 0.33–0.41], *p* < 0.0001). Therefore, the agreement in all three comparisons was fair, and more evident across clinical stages (Figure 3a,b). The contingency table displays how each single DD grade is reclassified by the different algorithms (Table A2).

### 3.5. Sorting the Undetermined DF: A Hierarchical Approach

We separately analyzed the undetermined DF resulting from ASE/EACVI and OH algorithms and ruled out S0HF subjects since Valsalva maneuver and PVF analysis were not performed. In the remaining 885 patients, Valsalva maneuver was interpretable in 93.3% and PVF in 96.5%, and AR_dur_-A_dur_ was measurable in 96.5% [1]. The large majority of undetermined DF in ASE/EACVI approaches resulted from the application of A-algorithm for SAHF patients. In each of the explored algorithms, high LAVi/a’ was significantly related to the severity of DD (Table 2).

In a stepwise regression model, LAVi/a’ was more significantly related to DD grades than a’ alone, which lost significance (*p* < 0.0001). Thus, LAVi/a’ was identified as the ideal parameter to reclassify undetermined DF. LAVi/a’ cut-off obtained by ROC analysis was 3.65 (AUC 0.91). Therefore, we tested the impact of different hierarchical combination of the usual supplementary DF parameters (AR_dur_–A_dur_ > 30 msec and mitral inflow during Valsalva maneuver), and LAVi/a’, on undetermined diastolic grade reclassification.

Among the 92 ASE/EACVI undetermined DF, 4 PVF (4%) and 8 Valsalva maneuvers (9%) were not feasible. We were able to reclassify 15 (16%) patients as DD using PVF, and 16 patients using Valsalva maneuver; 58 (63%) subjects were not upgraded. Adding LAVi/a’ to the previous parameters, 23 (25%) more undetermined DF were identified as DD, leaving 35 (38%) unclassified. We also tested LAVi/a’ alone, as the only reclassifying variable, detecting DD in 41 (45%) of 92 the undetermined DF patients.

Using OH algorithm, we identified 118 SAHF and SBHF as undetermined DF. PVF and Valsalva maneuver were not feasible in 6 (5%) and 21 (18%) individuals, respectively. LAVi/a’ was not measurable in only 1 of these subjects. PFV and Valsalva maneuver reclassified 21 (18%) and 17 (14%) individuals, respectively. The addition of LAVi/a’ led to the reclassification of 33 (28%) more patients, resulting in a total of 60% upgraded patients. Finally, assessing the undetermined DF by LAVi/a’ alone, successfully reclassified 63 (53%) subjects.

## 4. Discussion

In community-based cardiology service, LV DD is common, and it is associated with adverse cardiovascular outcome. Therefore, accurate detection and grading is of particular importance, particularly before overt HF develops. For this objective, non-invasive Doppler parameters are the corner stone management of such individuals [23,24]. Since protocols alternative to current guidelines have been proposed, the assessment of their agreement is key for their use in clinical practice. Discrepancies, in fact, importantly affect the clinical framing and follow-up activities.

### 4.1. Present Findings

The present study compares the comprehensive DF ASE/EACVI guidelines [5] with 2 alternative algorithms [7,8], in a substantial population of outpatients, mostly with preclinical HF. Our findings show that significant discrepancies among the 3 different criteria exist, resulting in only fair concordance on interpretation, with reclassification rates ranging from 30% to 37%. The discrepancies among different methods are mostly driven by the large prevalence of moderate to severe DD by JOHANSEN compared with the increased prevalence of normal DF to mild DD by ASE/EACVI. Noteworthy, differences in grading DD follow a gradient along with the characteristics of the 3 clinical stages, regarding few S0HF individuals and becoming progressively more pronounced in SAHF and SBHF (Figure 3a,b). DD could not be classified by ASE/EACVI and OH in a minority of individuals. However, we have demonstrated that a systematic use of feasible standard echocardiographic measurements (PVF and Valsalva maneuver) could solve such dilemma in about 60% of subjects. As a novel finding, our results show that the use of the ratio LAVi/a’, a rapid marker coupling morphological and functional characteristics of LA, and mirroring grades of DD irrespective of the method used, might be applied as a single most powerful potential tool to disentangle the undetermined DF.

### 4.2. Inconsistencies, Discrepancies, and Their Implications

Although differently arranged within each protocol, all investigated methods included LAVi, e’ and E/e’. These different manifestations of subclinical dysfunction do not necessarily coincide and are differentially represented in patients at different levels of risk [25]. In particular, enlarged LAVi does not necessarily mirror DD. Indeed, our previously reported upper limit of normal LAVi in non-athletes (38 mL/m^2^) [12] exceeded that suggested by guidelines (34 mL/m^2^). Importantly, the World Alliance Societies of Echocardiography (WASE) Normal Values Study, recently confirmed our preliminary findings in 2008 normal subjects irrespective of gender, race, or country of origin [26]. Moreover, LAVi is positively associated with fitness level in healthy populations [12,27] and in patients from a preventive clinic [28].

We have previously shown a significant lack of harmony among the 2009 ASE/EACVI classification and other validated classifications [1,2,3,4]. Since then, ASE/EACVI guidelines have been substantially revised, resulting in significant differences when integrally applied (i.e., including the concept of “myocardial disease”) [29]. Indeed, comparing our present data with those originally obtained (i.e., SAHF and SBHF) [1], we could identify a significantly increased prevalence of normal DF and grade I DD, with a clear reduction in grade II and III DD [4]. Some potential limits or inconsistencies are evident when applying each of the investigated algorithms. Applying ASE/EACVI in SAHF resulted in no patient in Grade I DD, and in SBHF, no patient had normal DF, which was otherwise detected in 35% or 44% of SBHF by JOHANSEN and OH, respectively. Twenty-five per cent of SBHF patients could not be graded by OH using basal assessment. If moderate or severe DD were included as criteria to SBHF, as previously suggested [30], adopting JOHANSEN would upgrade 98 SAHF patients (15%) to SBHF. Using both ASE/EACVI and OH would result in upgrading only 1 or 2% of SAHF patients to SBHF, respectively. Similarly, if increased E/e’ > 14 should be used, only 10 (2%) SAHF individuals would be reclassified to SBHF.

### 4.3. Sorting the Undetermined DF

While JOHANSEN algorithm allowed grading all patients, ASE/EACVI and OH failed to do so in about 10% individuals. Our data incidentally showed that using the updated ASE/EACVI results into a significant reduction of patients with undetermined DF grading in comparison with the 2009 version [4], consistent with Sorrentino et al. [29]. The main difference from that study, however, is that we used Valsalva maneuver and PVF analysis in all patients but S0HF individuals. We believe this is a novel contribution. The herein proposed hierarchical approach to undetermined DF was similarly effective both for ASE/EACVI and for OH. Besides the well-known Valsalva maneuver and quantitative PVF assessment, we introduced LAVi/a’, which resulted in an increased rate of upgrading patients with undetermined DD [13,14,15].

The a’ velocity is a fast and accurate marker of atrial systolic function and correlates with other measures of LA function [20,21,22,31]. Preclinical atrial dysfunction is characterized by reduced reservoir and conduit function, while atrial contractile function remains normal. As further deterioration of LV compliance occurs, a’ reduces, and LAVi progressively increases making LAVi/a’ a likely candidate for detecting raised LVFP [15,32,33]. Noteworthy, we have demonstrated that high LAVi/a’ values are directly related to E/e’ in SAHF and SBHF and to mirror worsening DD, independent of the utilized algorithm. We have consistently shown LAVi/a’ to be the most effective, single parameter, in sorting the undetermined DF and detecting raised LVFP. Advanced technologies have been proposed for better definition of DF by directly studying LA function [18,33]. However, they are not commonly available in primary care settings or may be cumbersome for routine daily practice. Thus, considering LAVi/a’ availability and feasibility in most echocardiographs, it makes it an operator friendly in the primary care practice.

### 4.4. Study Limitations

We acknowledge that our study has multiple limitations beyond its retrospective design. Our study cohort is free from patients with valvular heart disease or with HF. Thus, our findings cannot be extrapolated to those clinical conditions. JOHANSEN and ASE/EACVI have been compared in HF with reduced EF (HFrEF). Concordance between the algorithms was moderate, and the reclassification rate was 33% with comparable prognostic performance between the two algorithms by C-statistics [34]. Noteworthy, when comparing DDF Grade II vs. Grade III, only the JOHANSEN algorithm yielded prognostic information. Moreover, in a recent cohort of HFrEF, guideline-based diastolic-grading algorithm independently predicted mortality but was undefinable in 30% of subjects implicating a better prognostic performance of elevated E/e′ ratio [35]. However, in patients with normal LVEF, the relationship of E/e’ with LVFP is modest, and hence, using E/e’ for estimating LVFP in routine clinical practice is of limited benefit [36,37]. We used only un-enhanced TR velocities; thus, we cannot rule out some underestimation of the prevalence of increased estimated pulmonary pressures. Nonetheless, we used a comprehensive guideline-based [17] approach when TR quality was unsatisfactory, thus making this underestimation quite unlikely. Finally, the lack of invasive assessment may hinder the evaluation of the accuracy of our findings regarding LAVi/a’ versus cardiac catheterization. However, the inverse correlation of TDI-a’ with LVFP has been previously demonstrated, as well as its relationship with worse DD grades [5,20,21,22]. Thus, though future invasive studies focused on the relationship between LAVi/a’ and LVFP would be welcome, we do not think their absence may flaw our data or their interpretation.

## 5. Conclusions

Assessment of DF in primary-care outpatients free from HF according to guidelines-based or alternative protocols results in significant reclassification rates and only fair concordance. Subsequent discrepancies depend on the clinical profile of patients, being more evident in those with than without structural heart diseases. These findings may have important implications in demonstrating that different protocols cannot be utilized interchangeably neither in clinical practice nor in research activity. Future, possibly prospective, studies are thus needed to ascertain whether these inconsistencies affect prognostic stratification based in DD [38]. In patients with undetermined DF, adjunctive consolidated standard Doppler techniques (i.e., PVF analysis and Valsalva maneuver) should be pursued. A simple, rapid parameter integrating morphometric and functional information about LA, i.e., LAVi/a’, looks promising, although future studies should define its prognostic relevance in assessing and grading DD.

## Figures and Tables

**Table 1:** Clinical and echocardiographic characteristics of overall population and according to Heart Failure Stage 0, A and B (S0HF, SAHF, SBHF).

	Overall (*n* = 1158)	S0HF (*n* = 273)	SAHF (*n* = 644)	SBHF (*n* = 241)	*p*-Value
Male gender (*n*, %)	617 (53%)	162 (59%)	354 (55%)	101 (42%)	0.0002
Age (years)	58.4 ± 12.9	50.4 ± 11.0	58.8 ± 12.1	66.4 ± 11.4	<0.0001
Height (cm)	166.7 ± 10.0	170.2 ± 8.6	166.6 ± 10.0	162.8 ± 10.1	<0.0001
Weight (kg)	74.6 ± 14.4	70.9 ± 11.5	75.2 ± 15.1	77.0 ± 15.0	<0.0001
BMI (kg/m^2^)	26.8 ± 4.3	24.4 ± 3.2	27.0 ± 4.2	29.0 ± 4.5	<0.0001
BSA (m^2^)	1.83 ± 0.21	1.82 ± 0.18	1.83 ± 0.22	1.82 ± 0.21	0.5
Heart rate (bpm)	69.1 ± 11.2	71.1 ± 12.1	68.9 ± 10.6	67.3 ± 11.2	0.0005
Systolic Blood Pressure (mmHg)	138.6 ± 19.3	127.1 ± 12.9	139.6 ± 18.2	149.2 ± 21.6	<0.0001
Diastolic Blood Pressure (mmHg)	80.3 ± 8.6	77.7 ± 7.4	80.7 ± 8.4	81.8 ± 9.5	<0.0001
Left Ventricular Mass index (g/m^2^)	90.0 ± 22.4	81.5 ± 17.5	83.5 ± 15.1	116.8 ± 23.1	<0.0001
Left Ventricular End Diastolic Volume index (mL/m^2^)	58.1 ± 13.3	60.3 ± 13.2	54.8 ± 10.7	64.7 ± 16.4	<0.0001
Left Ventricular End Systolic Volume index (mL/m^2^)	20.1 ± 7.3	19.6 ± 5.1	18.6 ± 4.7	24.3 ± 11.8	<0.0001
LV ejection fraction (%)	65.7 ± 6.3	67.4 ± 4.7	65.9 ± 5.5	63.0 ± 8.8	<0.0001
LAVi (cm/m^2^)	33.4 ± 10.7	32.1 ± 9.0	31.6 ± 9.7	39.7 ± 12.2	<0.0001
E velocity (m/s)	0.76 ± 0.17	0.79 ± 0.14	0.75 ± 0.17	0.76 ± 0.20	0.001
A velocity (m/s)	0.71 ± 0.23	0.63 ± 0.15	0.71 ± 0.25	0.79 ± 0.22	<0.0001
A duration (ms)	124.1 ± 22.0	-	123.9 ± 22.3	124.7 ± 21.1	0.6
E/A ratio	1.22 ± 0.81	1.32 ± 0.39	1.22 ± 0.94	1.09 ± 0.73	0.004
Deceleration time E (ms)	206.1 ± 56.5	187.5 ± 44.5	208.4 ± 52.3	221.3 ± 71.8	<0.0001
Average s’ (cm/s)	9.6 ± 1.9	10.7 ± 1.9	9.5 ± 1.7	8.5 ± 1.7	<0.0001
Average e’ (cm/s)	10.7 ± 3.1	13.1 ± 2.8	10.6 ± 2.8	8.3 ± 2.3	<0.0001
Average a’ (cm/s)	11.0 ± 2.2	10.7 ± 2.0	11.2 ± 2.1	10.6 ± 2.4	0.0001
Average E/e’ ratio	7.6 ± 2.7	6.3 ± 1.3	7.4 ± 2.3	9.7 ± 3.6	<0.0001
LAVi/a’ ratio	3.23 ± 1.62	3.10 ± 1.15	2.93 ± 1.19	4.16 ± 2.54	<0.0001

Abbreviations: BMI, Body Mass Index; BSA, Body Surface Area; LV, Left Ventricular; LAVi, Left Atrial Volume index; E/A, peak early to late mitral diastolic velocities ratio; s’, peak annular systolic velocity; e’, peak early annular diastolic velocity; a’, peak late annular diastolic velocity; E/e’, ratio between E wave velocity and early diastolic velocity of the mitral annulus; LAVi/a’, ratio between Left Atrial Volume index and peak late annular diastolic velocity.

**Table 2:** Values of LAVi/a’ for grades of diastolic dysfunction according to different algorithms.

	Grade 0	Grade 1	Grade 2	Grade 3	*p*-Value
**ASE/EACVI**	2.86 ± 1.07	3.87 ± 2.35	5.22 ± 2.08	7.00 ± 3.94	<0.0001
**OH**	3.12 ± 1.52	2.49 ± 0.80	5.28 ± 2.04	8.25 ± 5.39	<0.0001
**JOHANSEN**	3.00 ± 1.15	2.41 ± 0.58	4.29 ± 2.36	5.59 ± 2.65	<0.0001

## Appendix A

**Table A1:** Specific clinical characteristics of overall population and according to Heart Failure Stages A and B (SAHF, SBHF).

	Overall (*n* = 885)	SAHF (*n* = 644)	SBHF (*n* = 241)	*p*-Value
Arterial Hypertension (*n*,%)	569 (64%)	377 (59%)	192 (80%)	<0.0001
Diabetes (*n*,%)	125 (14%)	69 (11%)	56 (23%)	<0.0001
Dyslipidemia (*n*,%)	377 (43%)	251 (39%)	126 (43%)	<0.0001
Smoke (*n*,%)	112 (13%)	85 (13%)	27 (11%)	0.4
CAD (*n*,%)	66 (7%)	25 (4%)	41 (17%)	<0.0001
CKD (*n*,%)	25 (3%)	11 (2%)	14 (6%)	0.002
Dyalisis (*n*,%)	6 (0.7%)	1 (0.1%)	5 (2%)	0.004
Tyroid disease (*n*,%)	88 (10%)	60 (9%)	28 (12%)	0.4
PAD (*n*,%)	72 (8%)	40 (6%)	32 (13%)	0.001
Other (*n*,%)	157 (18%)	107 (17%)	50 (21%)	0.2
ACEi (*n*,%)	247 (28%)	155 (24%)	92 (38%)	<0.0001
ARB (*n*,%)	205 (23%)	126 (20%)	79 (33%)	<0.0001
Beta blockers (*n*,%)	197 (22%)	121 (19%)	76 (32%)	<0.0001
Calcium channel blockers (*n*,%)	151 (17%)	92 (14%)	59 (25%)	0.0006
Diuretics (*n*,%)	243 (27%)	143 (22%)	100 (42%)	<0.0001
Alpha blockers (*n*,%)	39 (5%)	24 (4%)	15 (6%)	0.1
Acetylsalicylic acid (*n*,%)	192 (22%)	106 (17%)	86 (36%)	<0.0001
Statins (*n*,%)	205 (23%)	114 (18%)	91 (38%)	<0.0001
Digoxin (*n*,%)	4 (0.4%)	1 (0.2%)	3 (1%)	0.05
Antiarrythmics (*n*,%)	27 (3%)	15 (2%)	12 (5%)	0.05
NTG (*n*,%)	23 (3%)	3 (0.5%)	20 (8%)	<0.0001

Abbreviations: CAD, Coronary Artery Disease; CKD, Chronic Kidney Disease; PAD, Peripheral Artery Disease; ACEi, Angiotensin Converting Enzyme Inhibitor; ARB, Angiotensin Receptor Blocker; NTG, Nitroglycerin.

**Table A2:** The number diastolic grades conferred using (**a**) ASE/EACVI and JOHANSEN, (**b**) ASE/EACVI and OH, and (**c**) OH and JOHANSEN.

(a)						
	JOHANSEN					
ASE/EACVI	Normal	Grade 1	Grade 2	Grade 3	Total	
Normal	692 *	108	9	0	809	
Grade 1	82	35 *	85	7	209	
Grade 2	1	0	0	26	27	
Grade 3	2	0	1	8 *	11	
Indeterminate	10	0	86	6	102	
Total	787	143	181	47	1158	
**(b)**						
	**OH**					
**ASE/EACVI**	**Normal**	**Grade 1**	**Grade 2**	**Grade 3**	**Indeterminate**	**Total**
Normal	692 *	110	0	0	7	809
Grade 1	100	38 *	15	0	56	209
Grade 2	1	0	22 *	0	4	27
Grade 3	6	0	0	2 *	3	11
Indeterminate	30	9	7	1	55 *	101
Total	829	157	44	3	125	1158
**(c)**						
	**JOHANSEN**					
**OH**	**Normal**	**Grade 1**	**Grade 2**	**Grade 3**	**Total**	
Normal	708 *	69	46	6	829	
Grade 1	71	71 *	15	0	157	
Grade 2	0	0	16*	28	44	
Grade 3	0	0	0	3 *	3	
Indeterminate	8	3	104	10	125	
Total	787	143	181	47	1158	

* Values in agreement between the two algorithms.
